# Rapid Screening and Quantitative Analysis of 74 Pesticide Residues in Herb by Retention Index Combined with GC-QQQ-MS/MS

**DOI:** 10.1155/2021/8816854

**Published:** 2021-01-16

**Authors:** Peng Tan, Li Xu, Xi-Chuan Wei, Hao-Zhou Huang, Ding-Kun Zhang, Chen-Juan Zeng, Fu-Neng Geng, Xiao-Ming Bao, Hua Hua, Jun-Ning Zhao

**Affiliations:** ^1^College of Pharmacy, Chengdu University of Traditional Chinese Medicine, Chengdu 611137, China; ^2^Sichuan Academy of Traditional Chinese Medicine, State Key Laboratory of Quality Evaluation of Traditional Chinese Medicine, Chengdu 610041, China; ^3^Sichuan Key Laboratory for Medicinal American Cockroach, Sichuan Good Doctor Panxi Pharmaceutical Co.,Ltd., Chengdu 610000, China; ^4^Shimadzu Enterprise Management (China) Co.,Ltd., Chengdu 610023, China

## Abstract

In this research, a very practical QuEChERS-GC-MS/MS analytical approach for 74 pesticide residues in herb based on retention index was established. This novel analytical approach has two important technical advantages. One advantage is to quickly screen pesticide compounds in herbs without having to use a large number of pesticide standard substances at the beginning of the experiment. The other advantage is to assist in identifying the target pesticide compound accurately. A total of 74 kinds of pesticides were quickly prescreened in all *chuanxiong rhizoma* samples. The results showed that three kinds of pesticides were screened out in all the samples, including chlorpyrifos, fipronil, and procymidone, and the three pesticides were qualitatively and quantitatively determined. The RSD values for interday and intraday variation were acquired to evaluate the precision of the analytical approach, and the overall interday and intraday variations are not more than 1.97% and 3.82%, respectively. The variations of concentrations of the analyzed three pesticide compounds in sample CX16 are 0.74%–4.15%, indicating that the three pesticides in the sample solutions were stable in 48 h. The spiked recoveries of the three pesticides are 95.22%, 93.03%, and 94.31%, and the RSDs are less than ± 6.0%. The methodological verification results indicated the good reliability and accuracy of the new analytical method. This research work is a new application of retention index, and it will be a valuable tool to assist quickly and accurately in the qualitative and quantitative analysis of multipesticide residues in herbs.

## 1. Introduction

With more and more traditional herbal medicines playing an important role in protecting people's health, people are paying more attention to pesticide residues in herbal medicines [1, 2]. At present, most of the analytical methods for quantitative analysis of multipesticide residues are performed with a gas chromatography-triple quadrupole mass spectrometry (GC-MS/MS) or liquid chromatography-triple quadrupole mass spectrometry (LC-MS/MS) [3–5], which are usually applied to the quantitative determination of multipesticide residues in different fruits [6], honey [7], edible oils [8], tea [9, 10], and so on. In addition, there are some simple and novel pesticide residue analysis methods, such as high-performance liquid chromatography [11], multicolor nitrogen dots [12], and bioactive microfluidic paper device [13]. Unfortunately, the rapid screen and quantitative analysis of multipesticide residues in herbal medicines are still facing two very difficult problems. One difficulty is how to rapidly screen out the existing pesticide compounds in samples without the standard solution. Usually, a large number of pesticide standard substances were consumed during the quantitative analysis, resulting in high analysis costs. Another difficulty is how to accurately identify the target pesticide compound under the interference from complex chemical components in herbs. Because there are many impurities in herbs that seriously interfere with the target pesticide compounds, which makes it difficult to accurately identify the target pesticide compounds, in particular, some pyrethroid pesticides have optical isomers or cis/transisomers, such as cypermethrin and cyfluthrin, with up to 4 isomers, but it is actually difficult to obtain high-purity standard substances, which make it difficult to accurately identify them. Now, pharmacopoeia standards in many countries strictly require pesticide residue limits in herbal medicines, it is necessary to develop some rapid, high-sensitivity, low-cost analysis approach for multipesticide residues in herbs.

In the field of gas chromatography analysis, the retention index is an interesting concept with many important uses [14]. The retention index can assist in identifying target compounds by comparing the experimentally measured retention indices with known indices values [15, 16]. Now, there are many research reports about the retention index, such as petrochemicals analysis and identification of unknown compounds [17–20]. In the preliminary research of our research group, the retention index has been successfully applied to the analysis of 130 pesticide residues in *Panacis quinquefolii radix* in order to comply with Hong Kong's drug standards [21].

*Chuanxiong rhizoma* (Chuanxiong in Chinese) is a very famous traditional herb medicine; it has significant pharmacological effects such as inhibiting platelet activation [22], being anti-inflammatory [23, 24], and protecting brain damage [25, 26], which has many clinical applications. However, there are no reports on the multipesticide residues in Chuanxiong. In this study, the principle of the retention index was used to quickly prescreening multipesticide residues in samples without having to use large numbers of pesticide standard substances at the beginning of the experiment and assist accurately in identifying the target pesticide compounds, which provides a valuable analytical approach of multipesticide residues in traditional herbal medicines.

## 2. Experimental

### 2.1. Chemicals and Materials

During this exemplary application experiment, a total of 74 pesticide standard substances were used to verify the accuracy of the predicted retention times, including 31 different organophosphorus pesticide compounds, 27 different organochlorine pesticide compounds, and 16 different pyrethroid pesticide compounds, used for the quantitative analysis, and all of these pesticide standard substances were purchased from Neochema GmbH (100 *μ*g/mL, Germany). Fenthion-*d*6 was used as an internal standard compound, purchased from Dr. Ehrenstorfer GmbH (100 *μ*g/mL, Germany). The mass concentration of n-alkanes mixed standard solution was 1000 mg/L, purchased from o2si smart solutions company (C9∼C33, batch number 110219-06, USA). Acetonitrile and acetic acid for sample solution preparation were HPLC grade (Merck, Germany). QuEChERS extract tubes (batch number 6356241-01) were purchased from Agilent Technologies. All other chemical reagents were purchased from Sigma–Aldrich, China. A total of 40 batches of *chuanxiong rhizoma* herb samples were collected from Sichuan provinces, China.

### 2.2. Apparatus and Analytical Conditions

The chromatography assay was performed on a GC-MS/MS TQ8050 system equipped with an auto sample manager (Shimadzu, Japan). The chromatographic separation was performed on a Shimadzu SH-Rxi-5Sil MS column, 30 m × 0.25 mm × 0.25 *μ*m. The pressure value for high-pressure injection was 250 kPa, and the injection port temperature was set to 250°C; the injection mode was splitless. The high-purity helium was used as a carrier gas. The column flow rate was set to 1.69 mL/min, the linear velocity was set to 47.2 cm/s, and the purge flow rate was set to 5 mL/min. The temperature program of the column was set as follows: maintaining the initial temperature at 50°C for 1 minute, first increasing the temperature to 125°C at a rate of 25°C per minute, then increasing the temperature to 300°C at a rate of 10°C per minute, and finally holding for 15 minutes; the column equilibration time was set to 2 minutes. The injection volume of each sample test solution was 1.0 *μ*L. The detector was triple the quadrupole mass spectrometer, the ionization source was electron ionization (EI), the source temperature was set to 230°C, the high-purity argon was used as collision gas, and the MS transfer line temperature was set to 280°C. The monitoring mode was set to multiple reaction monitoring (MRM), and all of the monitoring ion pairs and collision energy (CE) were optimized and selected for subsequent analysis (see Table 1). In this experiment, time segmentation detection was applied to increase the analysis sensitivity.

### 2.3. Solutions Preparation Procedure

#### 2.3.1. Preparation of Mixed Standard Stock Solution

Measure accurately appropriate amount of each of the standard stock solutions, dilute with acetonitrile containing 0.05% acetic acid to prepare solutions containing 100 *μ*g/L and 1000 *μ*g/L.

#### 2.3.2. Preparation of Internal Standard Solution

Measure accurately appropriate amount of internal standard stock solution, diluted with acetonitrile to prepare a solution containing 5 *μ*g/mL.

#### 2.3.3. Preparation of Matrix-Matched Standard Solution

Weigh accurately 3.0 g of blank sample in sextuplicate, prepare in the same manner of sample test solution till “blowing to 0.4 mL with nitrogen gas at 40°C, adding accurately 50 *μ*L, 100 *μ*L of mixed standard stock solution (100 *μ*g/L) and 50 *μ*L, 100 *μ*L, 200 *μ*L, 400 *μ*L of mixed standard stock solution (1000 *μ*g/L)” [21], and dilute to 1 mL with acetonitrile, vortex, and filter (0.22 *μ*m).

#### 2.3.4. Preparation of Sample Test Solution

Prepare the sample solution according to the QuEChERS operating procedures and literature report [21].

### 2.4. Samples Assay Procedure

Firstly, the GC-MS/MS system automatically and accurately absorbed 1.0 *μ*L (5.0 *μ*g/mL) of n-alkanes C9∼C33 mixed standard solution for analysis under the given monitoring conditions, and then we have confirmed that all chromatographic peaks of C9∼C33 were correct; next, according to the retention indexes and retention times in the Smart Pesticides Database, the GC-MS/MS system automatically calculated the all predicted retention times of 74 target pesticide compounds. Then, the GC-MS/MS system automatically absorbed 1.0 *μ*L of each sample test solution and injected them into the GC-MS/MS for analysis. According to the experimental results, we prescreened which pesticides are contained in all samples using mass-to-charge ratios (*m*/*z*) and predicted retention times. Finally, we have prepared a mixed standard solution containing these screened out pesticides for targeted quantitative determination using the internal standard curve method.

### 2.5. Method Validation

The purpose of the validation of an analytical method is to ensure that the adopted method meets the requirements of the intended analytical applications. This newly established method was carried out in accordance with the international conference on harmonization guidelines (ICH Q2B, validation of analytical procedures, methodology).

#### 2.5.1. Precision

Intermediate-precision and repeatability were designed to evaluate the effect of random variable factors on precision. The variable factors include different dates and different analysts. The relative standard deviation (RSD) was used to evaluate the variation range of the results. Intraday and interday repeatability was determined by six-replicate analyses of sample CX16 within one and two consecutive days, respectively. In a specified range, use results from 6 test samples at the same concentration to evaluate the repeatability of the precision study.

#### 2.5.2. Linearity

The linearity of this new analytical method is its ability to elicit test results that are directly proportional to the concentration of the analyte in samples within a given range. The samples with varying concentrations of analytes for linearity determination are prepared by diluting accurately a stock solution. During the experiment, 6 portions of sample solutions were prepared. The regression equation and correlation coefficient were used to evaluate the correlation between the peak area (*y*) and the injection mass concentration (*x*, *μ*g/L) of each pesticide compound.

#### 2.5.3. Sensitivity

The limit of detection (LOD) was defined as the lowest mass concentration of each pesticide compound resulting in a signal-to-noise ratio of 3 : 1. The limit of quantification (LOQ) was defined as the lowest mass concentration of each pesticide compound resulting in a signal-to-noise ratio of 10 : 1.

#### 2.5.4. Extraction Accuracy

Accuracy was usually represented as percent recovery and was determined in the specified range. High-purity standard substances were used for the analysis of the recovery of the added sample. In this experiment, a certain number of the standard substances of the test substances were precisely added into the test sample with known content of analyte to be examined. The recovery ratio was calculated by the margin of the determined value and the number of the substances being examined divided by the number of the added standard substances:

(1) $$\begin{array}{c} \mathrm{recovery}\;\left(\%\right)=\frac{\left(C-A\right)}{B}\times 100\text{\%,} \end{array}$$

where *A* is the amount of the analyte in the substance being examined; *B* is the amount of the added standard substance; and *C* is the determined value.

#### 2.5.5. Stability

The stability of the analyzed pesticide compounds in the sample solution was detected by analyzing sample CX16, and the peak areas of the analyzed pesticide compounds at 0, 2, 4, 8, 24, and 48 h were recorded. Variations in the content were expressed as RSD values.

## 3. Results and Discussion

### 3.1. Obtained Predicted Retention Times by Retention Indexes of n-Alkanes C9∼C33

**Figure 1:**
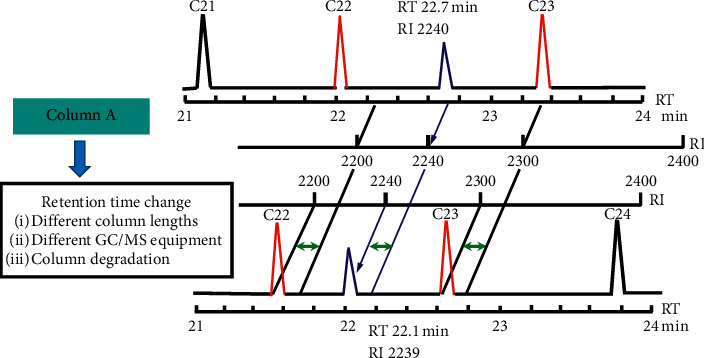
Schematic diagram of predicting retention time of target pesticide using retention index principle.

**Figure 2:**
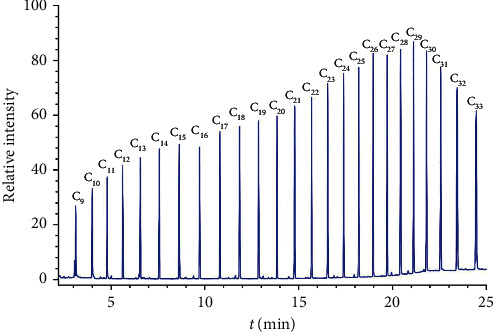
Typical total ionic chromatogram (TIC) of n-alkanes C9∼C33 mixed standard solution.

Experiment results showed that the first target pesticide compound was detected at 6 minutes (dichlorvos), and the last target pesticide compound was detected at 22 minutes (deltamethrin-2). The actual measured retention indexes of 74 target pesticide compounds are displayed in Table 2, and as shown, there is a small difference between the actual measured retention indexes and the original retention indexes listed in the Smart Pesticides Database. At the same time, the predicted retention times of 74 target pesticides were obtained by actual measured retention indexes of 74 pesticide compounds combined with the Smart Pesticides Database (see Table 2). The schematic diagram of predicting retention time of target pesticide using retention index principle is displayed Figure 1. The typical total ionic chromatogram of C9∼C33 mixed standard solution is displayed in Figure 2.

### 3.2. Verification Accuracy of the Predicted Retention Times Using Standard Substances

In order to verify the accuracy of the predicted retention times, a mixed standard substances solution containing 74 pesticide standard substances was analyzed under the same analysis conditions. The results showed that all the predicted retention times were very close to the actual measured retention times; it was worth noting that all the times deviations were within 0.02 min, and this means that these predicted retention times were very accurate. The detailed data are summarized and displayed in Table 2.

### 3.3. Rapid Prescreening 74 Pesticide Compounds Using Predicted Retention Times

One important application of the predicted retention times was to quickly screen out pesticide compounds in herbs without having to use a large number of pesticide standard substances at the beginning of the experiment. All 40 batches of *chuanxiong rhizoma* samples have been quickly screened using the predicted retention times of 74 target pesticides and mass-to-charge ratios. The prescreened results showed that all samples only contained three different pesticide compounds, including chlorpyrifos, fipronil, and procymidone. Because this experiment was an exemplary research, in order to confirm the accuracy of the screening results, 74 pesticide standard substances were used to verify the accuracy of the screening results. The confirmation results also showed that all samples contained the three pesticides. Next, it was very simple and convenient to quantitatively analyze the three pesticide compounds that have been screened out. More importantly, this experiment has demonstrated that the predicted retention time obtained by the retention index principle could be used to screen out pesticides in herbs without having to use large amounts of pesticide standard substances.

### 3.4. Assist in Identifying Target Pesticide Compounds Using Predicted Retention Times

**Figure 3:**
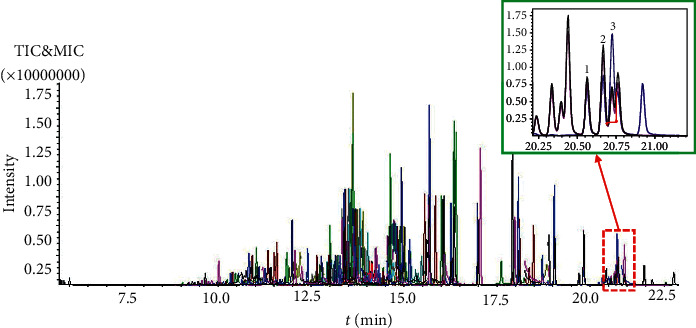
Typical total ionic chromatogram of mixed chuanxiong rhizoma sample solution containing 74 pesticide standard substances (200 *μ*g/L).

Another practical and important application of the predicted retention time was to assist accurately in identifying the target pesticide compound when the target pesticide compound was seriously disturbed. In cases where the mass spectrometry system cannot automatically identify or misidentify them, the predicted retention time of the target pesticide compound can be referenced to help in the qualitative analysis. Taking cypermethrin-3 as an example, we want to accurately identify and qualify cypermethrin-3 in mass chromatogram, but cypermethrin-2 and cypermethrin-4 were also located near cypermethrin-3, the actual measured retention times of the three isomers were 20.561, 20.663, and 20.717 min, the predicted retention times of the three isomers were 20.557, 20.662, and 20.711 min, and the deviations were 0.004, 0.001, and 0.006 min, respectively. When the mass spectrometry system could not accurately identify cypermethrin-3 automatically, or in the absence of a cypermethrin-3 monomer standard solution, the predicted retention times of the three isomers of cypermethrin were very useful and could assist in identifying cypermethrin-3, the detailed information is displayed in Figure 3 and Table 2. This analytical approach can assist in identifying other difficult-to-identify target pesticide compounds.

### 3.5. Samples Assay Result

**Figure 4:**
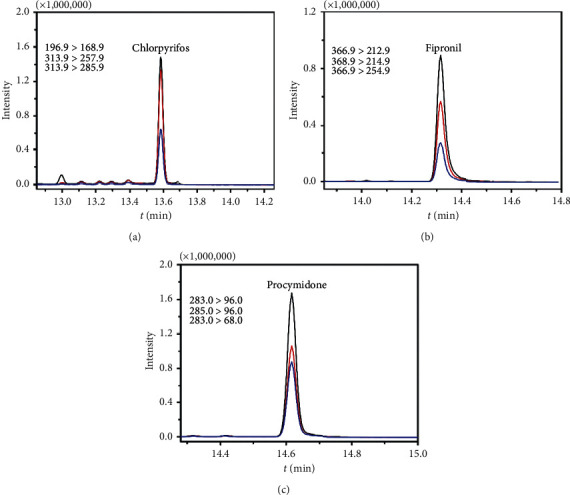
Typical mass chromatograms of chlorpyrifos, fipronil, and procymidone in *chuanxiong rhizoma* sample.

The developed GC-MS/MS analytical method was applied to quickly screen out and assist in the quantitative determination of the multipesticide residues in *chuanxiong rhizoma* samples; the identification of all pesticide compounds was based on mass-to-charge ratios and predicted retention times. The quick screening results showed all the samples containing three different pesticide compounds, including chlorpyrifos, fipronil, and procymidone. Therefore, the next step was to target quantifying the three pesticides using corresponding standard solution. The quantitative assay results of 40 batches of samples showed that only one batch detected chlorpyrifos at a level of 485.92 *μ*g/kg. Four batches of samples were detected with fipronil in the range of 28.62 *μ*g/kg to 193.12 *μ*g/kg. It was worth noting that a total of 29 batches of samples detected procymidone, and the content of procymidone in CX19 was almost 131-fold higher than this in CX10 (3712.16 vs. 28.25 *μ*g/kg). This result indicated that the use of procymidone in the planting process of *chuanxiong rhizoma* has become more common, and the residue amount of procymidone in *Ligusticum chuanxiong* Hort was very serious. Therefore, it is necessary to continuously monitor the residual amount of procymidone in *chuanxiong rhizoma* to ensure the safety of the clinical use of this herb. Typical mass chromatograms of procymidone, chlorpyrifos, and fipronil in *chuanxiong rhizoma* samples solution are displayed in Figure 4. The detailed results are summarized in Table 3.

### 3.6. Validation of the GC-MS/MS Method

During the experiment, linearity, precision, sensitivity, stability, and accuracy analyses were completed to evaluate the newly established analysis approach. Linear regression equations of the three target pesticide compounds were acquired at six concentration levels in triplicate, and the LODs and LOQs of the three pesticides were optimized for subsequent analysis (see Table 4). The RSD values for interday and intraday variation were acquired to evaluate the precision of the analytical approach, and the overall inter- and intraday variations are not more than 1.97% and 3.82%, respectively (see Table 4). The variations of concentrations of the analyzed three pesticide compounds in sample CX16 are 0.74%–4.15%, indicating that the three pesticides in the sample solutions were maintained stable in 48 h. The spiked recoveries of the three pesticides are 95.22%, 93.03%, and 94.31%, and the RSDs were less than ±6.0%. The methodological verification results indicated the good reliability and accuracy of the new analytical method.

## 4. Conclusions

In this exemplary research, a very practical GC-MS/MS analytical approach for 74 pesticide residues in *chuanxiong rhizoma* herb based on the retention index was established. This novel analytical approach has two important applications. One was to rapidly screen out the multipesticides in herbal medicines without having to use a large number of standard substances at the beginning of the experiment. The other important application was to assist accurately in qualitative target pesticide compound. This new analytical approach can increase pesticide analysis efficiency and significantly reduce the cost of analysis. This research was a new application of the retention index, and it will be a valuable tool for quickly and accurately quantifying the analysis of multipesticide residues in traditional herbal medicines.

## Figures and Tables

**Table 1:** Monitoring ion pairs and collision energy of 74 pesticide compounds.

Number	Pesticide name	*m*/*z* 1	Collision energy (V)	*m*/*z* 2	Collision energy (V)
1	Dichlorvos	109.0 > 79.0	8	185.0 > 93.0	14
2	Tecnazene	260.9 > 202.9	14	202.9 > 142.9	22
3	Diphenylamine	169.1 > 66.0	24	167.1 > 139.1	28
4	Chlordimeform	196.0 > 181.0	10	181.0 > 140.0	15
5	Trifluralin	306.1 > 264.1	8	264.1 > 160.1	18
6	*α*-BHC	180.9 > 144.9	16	218.9 > 182.9	8
7	Hexachlorobenzene	283.8 > 248.8	24	283.8 > 213.8	28
8	Pentachloroanisole	264.8 > 236.8	16	279.9 > 236.8	26
9	Dicloran	206.0 > 176.0	10	176.0 > 148.0	12
10	*β*-BHC	180.9 > 144.9	16	218.9 > 182.9	8
11	Quintozene	264.8 > 236.8	10	294.8 > 236.8	15
12	*γ*-BHC	180.9 > 144.9	16	218.9 > 182.9	8
13	Terbufos	231.0 > 128.9	26	231.0 > 174.9	14
14	Chlorothalonil	263.9 > 168.0	24	263.9 > 228.8	18
15	Tefluthrin	177.0 > 127.1	16	177.0 > 137.1	16
16	*δ*-BHC	180.9 > 144.9	16	218.9 > 182.9	8
17	Pentachloraniline	262.9 > 191.9	22	264.9 > 193.9	18
18	Chlorpyrifos-methyl	285.9 > 93.0	22	287.9 > 93.0	22
19	Vinclozolin	212.0 > 172.0	16	285.0 > 212.0	12
20	Parathion-methyl	263.0 > 109.0	14	125.0 > 47.0	12
21	Heptachlor	271.8 > 236.9	20	273.8 > 238.9	16
22	Fenchlorphos	284.9 > 269.9	16	286.9 > 271.9	18
23	Octachlorodipropylether	130.0 > 95.0	20	181.0 > 85.0	10
24	Fenitrothion	283.1 > 115.0	18	283.1 > 131.0	18
25	Methyl-pentachlorophenyl sulfide	295.8 > 262.9	14	295.8 > 245.8	30
26	Dichlofluanid	223.9 > 123.1	8	167.1 > 124.1	10
27	Chlorpyrifos	196.9 > 168.9	14	313.9 > 257.9	14
28	Aldrin	262.9 > 191.0	34	262.9 > 193.0	28
29	Fenthion-d6	284.0 > 115.0	20	284.0 > 169.0	15
30	Chlorthal-dimethyl	298.9 > 220.9	24	300.9 > 222.9	26
31	Parathion-ethyl	139.0 > 109.0	8	291.1 > 109.0	14
32	Triadimefon	208.1 > 181.0	10	208.1 > 111.0	22
33	Dicofol	139.0 > 111.0	16	139.0 > 75.0	28
34	Butralin	266.1 > 190.1	12	266.1 > 236.1	8
35	Bromophos-methyl	330.9 > 315.9	14	328.9 > 313.9	18
36	Pendimethalin	252.1 > 162.1	10	252.1 > 191.1	8
37	Fipronil	366.9 > 212.9	30	368.9 > 214.9	30
38	Heptachlor exo-epoxide	352.8 > 262.9	14	354.8 > 264.9	20
39	Chlordane-oxy	185.0 > 149.0	6	185.0 > 121.0	12
40	Heptachlor endo-epoxide	352.8 > 253.0	26	354.8 > 253.0	18
41	Dimepiperate	119.1 > 91.1	10	145.1 > 112.1	8
42	Procymidone	283.0 > 96.0	10	285.0 > 96.0	10
43	Triadimenol-1	168.1 > 70.0	10	128.1 > 65.0	22
44	Triadimenol-2	168.1 > 70.0	10	128.1 > 65.0	22
45	Bromophos-ethyl	358.9 > 302.9	16	302.9 > 284.9	18
46	Chlordane-trans	374.8 > 265.9	26	372.8 > 263.9	28
47	o, p'-DDE	246.0 > 176.0	30	248.0 > 176.0	28
48	Flumetralin	143.0 > 107.0	21	143.0 > 83.0	18
49	Chlordane-cis	374.8 > 265.9	26	372.8 > 263.9	28
50	*α*-Endosulfan	194.9 > 160.0	8	194.9 > 125.0	24
51	p, p'-DDE	246.0 > 176.0	30	317.9 > 248.0	24
52	Dieldrin	276.9 > 241.0	8	262.9 > 193.0	34
53	o, p'-DDD	235.0 > 165.0	24	237.0 > 165.0	28
54	Chlorfenapyr	247.1 > 227.0	16	139.0 > 102.0	12
55	Nitrofen	202.0 > 139.0	24	282.9 > 253.0	12
56	Endrin	262.9 > 191.0	30	262.9 > 193.0	28
57	*β*-Endosulfan	194.9 > 160.0	8	194.9 > 125.0	24
58	p, p'-DDD	235.0 > 165.0	24	237.0 > 165.0	28
59	o, p'-DDT	235.0 > 165.0	24	237.0 > 165.0	28
60	Endosulfan sulfate	271.8 > 236.9	18	386.8 > 252.9	16
61	p, p'-DDT	235.0 > 165.0	24	237.0 > 165.0	28
62	Bifenthrin	181.1 > 166.1	12	181.1 > 179.1	12
63	Bromopropylate	340.9 > 182.9	18	340.9 > 184.9	20
64	Methoxychlor	227.1 > 169.1	24	227.1 > 212.1	14
65	Fenpropathrin	181.1 > 152.1	22	265.1 > 210.1	12
66	Phenothrin-1	123.1 > 81.0	8	183.1 > 153.1	14
67	Phenothrin-2	123.1 > 81.0	8	183.1 > 153.1	14
68	Cyhalothrin-1	208.0 > 181.0	8	197.0 > 141.0	12
69	Acrinathrin	181.1 > 152.1	26	289.1 > 93.0	14
70	Cyhalothrin-2	208.0 > 181.0	8	197.0 > 141.0	12
71	Mirex	271.8 > 236.8	18	273.8 > 238.8	18
72	Acrinathrin-2	181.1 > 152.1	26	289.1 > 93.0	14
73	Permethrin-1	183.1 > 153.1	14	183.1 > 168.1	14
74	Permethrin-2	183.1 > 153.1	14	183.1 > 168.1	14
75	Cyfluthrin-1	163.1 > 127.1	6	163.1 > 91.0	14
76	Cyfluthrin-2	163.1 > 127.1	6	163.1 > 91.0	14
77	Cyfluthrin-3	163.1 > 127.1	6	163.1 > 91.0	14
78	Cyfluthrin-4	163.1 > 127.1	6	163.1 > 91.0	14
79	Cypermethrin-1	163.1 > 127.1	6	163.1 > 91.0	14
80	Cypermethrin-2	163.1 > 127.1	6	163.1 > 91.0	14
81	Cypermethrin-3	163.1 > 127.1	6	163.1 > 91.0	14
82	Flucythrinate-1	199.1 > 157.1	10	157.1 > 107.1	12
83	Quizalofop-ethyl	372.1 > 299.1	14	299.1 > 255.1	18
84	Cypermethrin-4	163.1 > 127.1	6	163.1 > 91.0	14
85	Flucythrinate-2	199.1 > 157.1	10	157.1 > 107.1	12
86	Fenvalerate-1	225.1 > 119.1	20	225.1 > 147.1	10
87	Fenvalerate-2	225.1 > 119.1	20	225.1 > 147.1	10
88	Deltamethrin-1	180.9 > 151.9	22	252.9 > 93.0	20
89	Deltamethrin-2	180.9 > 151.9	22	252.9 > 93.0	20

Compound 29 is internal standard compound. Some pesticides have multiple retention times because of isomers.

**Table 2:** Results of the predicted retention times and the actual measured retention times.

Number	Pesticide name	Retention index from the database	Predicted retention time (min)	Actual measured retention index	Actual measured retention time (min)	Time deviation value (min)
1	Dichlorvos	1248	6.023	1244	6.017	0.006
2	Tecnazene	1595	9.618	1597	9.621	0.003
3	Diphenylamine	1631	10.007	1592	10.001	0.006
4	Chlordimeform	1660	10.319	1661	10.323	0.004
5	Trifluralin	1666	10.384	1666	10.380	0.004
6	*α*-BHC	1705	10.803	1707	10.820	0.017
7	Hexachlorobenzene	1710	10.856	1709	10.843	0.013
8	Pentachloroanisole	1723	10.993	1725	11.009	0.016
9	Dicloran	1731	11.077	1731	11.081	0.004
10	*β*-BHC	1755	11.329	1755	11.328	0.001
11	Quintozene	1759	11.371	1763	11.373	0.002
12	Lindane	1769	11.476	1755	11.468	0.008
13	Terbufos	1778	11.571	1778	11.571	0.000
14	Chlorothalonil	1803	11.833	1803	11.835	0.002
15	Tefluthrin	1819	11.996	1818	11.982	0.014
16	*δ*-BHC	1825	12.057	1825	12.055	0.002
17	Pentachloraniline	1855	12.363	1858	12.378	0.015
18	Chlorpyrifos-methyl	1882	12.638	1883	12.653	0.015
19	Vinclozolin	1891	12.730	1857	12.712	0.018
20	Parathion-methyl	1896	12.781	1895	12.770	0.011
21	Heptachlor	1909	12.910	1915	12.927	0.017
22	Fenchlorphos	1916	12.979	1918	12.995	0.016
23	Octachlorodipropylether	1932	13.136	1932	13.140	0.004
24	Fenitrothion	1946	13.273	1945	13.267	0.006
25	Methyl-pentachlorophenyl sulfide	1954	13.351	1957	13.365	0.014
26	Dichlofluanid	1959	13.400	1960	13.410	0.010
27	Chlorpyrifos	1977	13.577	1978	13.583	0.006
28	Aldrin	1981	13.601	1964	13.595	0.006
29	Fenthion-d6	1982	13.626	1981	13.616	0.010
30	Chlorthal-dimethyl	1986	13.665	1988	13.677	0.012
31	Parathion-ethyl	1993	13.734	1993	13.730	0.004
32	Triadimefon	1999	13.793	1999	13.793	0.000
33	Dicofol	2008	13.878	2009	13.889	0.011
34	Butralin	2012	13.935	2014	13.938	0.003
35	Bromophos-methyl	2021	14.012	2023	14.018	0.006
36	Pendimethalin	2044	14.227	2046	14.232	0.005
37	Fipronil	2055	14.321	2054	14.314	0.007
38	Heptachlor exo-epoxide	2061	14.397	2067	14.401	0.004
39	Chlordane-oxy	2061	14.398	2067	14.409	0.011
40	Heptachlor endo-epoxide	2069	14.453	2075	14.468	0.015
41	Dimepiperate	2086	14.613	2087	14.622	0.009
42	Procymidone	2086	14.613	2086	14.617	0.004
43	Triadimenol-1	2087	14.622	2086	14.617	0.005
44	Triadimenol-2	2103	14.772	2103	14.768	0.004
45	Bromophos-ethyl	2106	14.799	2109	14.812	0.013
46	Chlordane-trans	2110	14.835	2108	14.817	0.018
47	o, p'-DDE	2116	14.889	2120	14.905	0.016
48	Flumetralin	2127	14.989	2128	14.994	0.005
49	Chlordane-cis	2137	15.079	2144	15.081	0.002
50	*α*-Endosulfan	2139	15.098	2144	15.105	0.007
51	p, p'-DDE	2185	15.514	2188	15.524	0.010
52	Dieldrin	2195	15.604	2175	15.601	0.003
53	o, p'-DDD	2199	15.640	2224	15.655	0.015
54	Chlorfenapyr	2222	15.841	2222	15.837	0.004
55	Nitrofen	2239	15.969	2213	15.965	0.004
56	Endrin	2240	15.997	2247	16.011	0.014
57	*β*-Endosulfan	2266	16.223	2270	16.239	0.016
58	p, p'-DDD	2276	16.310	2299	16.312	0.002
59	o, p'-DDT	2280	16.345	2259	16.361	0.016
60	Endosulfan sulfate	2351	16.945	2355	16.958	0.013
61	p, p'-DDT	2359	17.032	2364	17.040	0.008
62	Bifenthrin	2469	17.909	2469	17.908	0.001
63	Bromopropylate	2475	17.957	2438	17.951	0.006
64	Methoxychlor	2487	18.043	2460	18.036	0.007
65	Fenpropathrin	2493	18.101	2492	18.096	0.005
66	Phenothrin-1	2526	18.359	2526	18.362	0.003
67	Phenothrin-2	2540	18.467	2541	18.473	0.006
68	Cyhalothrin-1	2573	18.722	2572	18.713	0.009
69	Acrinathrin	2591	18.861	2595	18.871	0.010
70	Cyhalothrin-2	2595	18.892	2595	18.891	0.001
71	Mirex	2610	19.005	2615	19.014	0.009
72	Acrinathrin-2	2619	19.062	2616	19.052	0.010
73	Permethrin-1	2702	19.696	2703	19.701	0.005
74	Permethrin-2	2720	19.821	2721	19.828	0.007
75	Cyfluthrin-1	2777	20.232	2778	20.236	0.004
76	Cyfluthrin-2	2791	20.333	2791	20.334	0.001
77	Cyfluthrin-3	2799	20.390	2800	20.395	0.005
78	Cyfluthrin-4	2806	20.439	2806	20.439	0.000
79	Cypermethrin-1	2823	20.557	2824	20.561	0.004
80	Cypermethrin-2	2838	20.662	2824	20.663	0.001
81	Cypermethrin-3	2845	20.711	2847	20.717	0.006
82	Flucythrinate-1	2847	20.724	2847	20.726	0.002
83	Quizalofop-ethy-l	2848	20.743	2851	20.751	0.008
84	Cypermethrin-4	2852	20.759	2852	20.762	0.003
85	Flucythrinate-2	2875	20.919	2875	20.922	0.003
86	Fenvalerate-1	2951	21.442	2930	21.448	0.006
87	Fenvalerate-2	2981	21.628	2930	21.619	0.009
88	Deltamethrin-1	3031	22.016	3034	22.024	0.008
89	Deltamethrin-2	3060	22.235	3063	22.242	0.007

**Table 3:** Targeted quantitative analysis results of three pesticides in 40 batches of samples (mean, *μ*g/kg).

Samples	Procymidone	Chlorpyrifos	Fipronil
CX01	94.77	ND	ND
CX02	47.11	ND	ND
CX03	60.75	ND	ND
CX04	42.89	ND	ND
CX05	29.91	ND	ND
CX06	71.73	ND	ND
CX07	49.85	ND	ND
CX08	31.75	ND	ND
CX09	ND	485.92	ND
CX10	28.25	ND	ND
CX11	56.13	ND	ND
CX12	56.95	ND	ND
CX13	2841.54	ND	ND
CX14	ND	ND	ND
CX15	69.50	ND	ND
CX16	159.29	ND	ND
CX17	35.61	ND	ND
CX18	ND	ND	28.62
CX19	3712.16	ND	ND
CX20	77.30	ND	ND
CX21	64.58	ND	ND
CX22	ND	ND	193.12
CX23	ND	ND	ND
CX24	61.62	ND	38.28
CX25	44.16	ND	ND
CX26	77.72	ND	ND
CX27	ND	ND	64.21
CX28	71.27	ND	ND
CX29	ND	ND	ND
CX30	142.19	ND	ND
CX31	66.31	ND	ND
CX32	81.09	ND	ND
CX33	ND	ND	ND
CX34	ND	ND	ND
CX35	57.36	ND	ND
CX36	82.61	ND	ND
CX37	ND	ND	ND
CX38	ND	ND	ND
CX39	85.36	ND	ND
CX40	96.77	ND	ND

ND represents that the limit of detection has not been reached.

**Table 4:** Results of methodological verification (*n* = 6).

Pesticides	Linear regression equation	*R* ^2^	LOD (*μ*g/L)	LOQ (*μ*g/L)	RSD for interday (%)	RSD for intraday (%)
Chlorpyrifos	*Y* = 402*X* + 3.55 × 10^3^	0.9991	0.0225	0.0964	1.97	2.98
Fipronil	*Y* = 291*X* − 1.38 × 10^3^	0.9996	0.0155	0.0565	1.44	3.17
Procymidone	*Y* = 594*X* + 2.91 × 10^3^	0.9993	0.0088	0.0354	1.63	3.82

## Data Availability

The underlying data supporting the results of our study can be found in the manuscript.

## Supplementary Materials

Supplementary Materials Table S1: monitoring ion pairs and collision energy of 74 pesticide compounds. Table S2: quantitative analysis methodological results of the three target pesticides compounds that were screened.
