# Supplementary material for: Rapid Screening and Quantitative Analysis of 74 Pesticide Residues in Herb by Retention Index Combined with GC-QQQ-MS/MS
Source: J Anal Methods Chem. 2021 Jan 16;2021:8816854. doi: 10.1155/2021/8816854 (PMC7826212; doi:10.1155/2021/8816854)
Supplement: Supplementary Materials — Table S1: monitoring ion pairs and collision energy of 74 pesticide compounds. Table S2: quantitative analysis methodological results of the three target pesticides compounds that were screened. [file 8816854.f1.docx]

**Table S1** Monitoring ion pairs and collision energy of 74 pesticide compounds.

| Number | Pesticide name | m/z 1 | Collision energy (V) | m/z 2 | Collision energy (V) |
| --- | --- | --- | --- | --- | --- |
| 1 | Dichlorvos | 109.0>79.0 | 8 | 185.0>93.0 | 14 |
| 2 | Tecnazene | 260.9>202.9 | 14 | 202.9>142.9 | 22 |
| 3 | Diphenylamine | 169.1>66.0 | 24 | 167.1>139.1 | 28 |
| 4 | Chlordimeform | 196.0>181.0 | 10 | 181.0>140.0 | 15 |
| 5 | Trifluralin | 306.1>264.1 | 8 | 264.1>160.1 | 18 |
| 6 | α-BHC | 180.9>144.9 | 16 | 218.9>182.9 | 8 |
| 7 | Hexachlorobenzene | 283.8>248.8 | 24 | 283.8>213.8 | 28 |
| 8 | Pentachloranisole | 264.8>236.8 | 16 | 279.9>236.8 | 26 |
| 9 | Dicloran | 206.0>176.0 | 10 | 176.0>148.0 | 12 |
| 10 | β-BHC | 180.9>144.9 | 16 | 218.9>182.9 | 8 |
| 11 | Quintozene | 264.8>236.8 | 10 | 294.8>236.8 | 15 |
| 12 | γ-BHC | 180.9>144.9 | 16 | 218.9>182.9 | 8 |
| 13 | Terbufos | 231.0>128.9 | 26 | 231.0>174.9 | 14 |
| 14 | Chlorothalonil | 263.9>168.0 | 24 | 263.9>228.8 | 18 |
| 15 | Tefluthrin | 177.0>127.1 | 16 | 177.0>137.1 | 16 |
| 16 | δ-BHC | 180.9>144.9 | 16 | 218.9>182.9 | 8 |
| 17 | Pentachloraniline | 262.9>191.9 | 22 | 264.9>193.9 | 18 |
| 18 | Chlorpyrifos-methyl | 285.9>93.0 | 22 | 287.9>93.0 | 22 |
| 19 | Vinclozolin | 212.0>172.0 | 16 | 285.0>212.0 | 12 |
| 20 | Parathion-methyl | 263.0>109.0 | 14 | 125.0>47.0 | 12 |
| 21 | Heptachlor | 271.8>236.9 | 20 | 273.8>238.9 | 16 |
| 22 | Fenchlorphos | 284.9>269.9 | 16 | 286.9>271.9 | 18 |
| 23 | Octachlorodipropylether | 130.0>95.0 | 20 | 181.0>85.0 | 10 |
| 24 | Fenitrothion | 283.1>115.0 | 18 | 283.1>131.0 | 18 |
| 25 | Methyl-pentachloropheny lsulfide | 295.8>262.9 | 14 | 295.8>245.8 | 30 |
| 26 | Dichlofluanid | 223.9>123.1 | 8 | 167.1>124.1 | 10 |
| 27 | Chlorpyrifos | 196.9>168.9 | 14 | 313.9>257.9 | 14 |
| 28 | Aldrin | 262.9>191.0 | 34 | 262.9>193.0 | 28 |
| 29 | Fenthion-d6 | 284.0>115.0 | 20 | 284.0>169.0 | 15 |
| 30 | Chlorthal-dimethyl | 298.9>220.9 | 24 | 300.9>222.9 | 26 |
| 31 | Parathion-ethyl | 139.0>109.0 | 8 | 291.1>109.0 | 14 |
| 32 | Triadimefon | 208.1>181.0 | 10 | 208.1>111.0 | 22 |
| 33 | Dicofol | 139.0>111.0 | 16 | 139.0>75.0 | 28 |
| 34 | Butralin | 266.1>190.1 | 12 | 266.1>236.1 | 8 |
| 35 | Bromophos-methyl | 330.9>315.9 | 14 | 328.9>313.9 | 18 |
| 36 | Pendimethalin | 252.1>162.1 | 10 | 252.1>191.1 | 8 |
| 37 | Fipronil | 366.9>212.9 | 30 | 368.9>214.9 | 30 |
| 38 | Heptachlor exo-epoxide | 352.8>262.9 | 14 | 354.8>264.9 | 20 |
| 39 | Chlordane-oxy | 185.0>149.0 | 6 | 185.0>121.0 | 12 |
| 40 | Heptachlor endo-epoxide | 352.8>253.0 | 26 | 354.8>253.0 | 18 |
| 41 | Dimepiperate | 119.1>91.1 | 10 | 145.1>112.1 | 8 |
| 42 | Procymidone | 283.0>96.0 | 10 | 285.0>96.0 | 10 |
| 43 | Triadimenol-1 | 168.1>70.0 | 10 | 128.1>65.0 | 22 |
| 44 | Triadimenol-2 | 168.1>70.0 | 10 | 128.1>65.0 | 22 |
| 45 | Bromophos-ethyl | 358.9>302.9 | 16 | 302.9>284.9 | 18 |
| 46 | Chlordane-trans | 374.8>265.9 | 26 | 372.8>263.9 | 28 |
| 47 | o, p’-DDE | 246.0>176.0 | 30 | 248.0>176.0 | 28 |
| 48 | Flumetralin | 143.0>107.0 | 21 | 143.0>83.0 | 18 |
| 49 | Chlordane-cis | 374.8>265.9 | 26 | 372.8>263.9 | 28 |
| 50 | α-Endosulfan | 194.9>160.0 | 8 | 194.9>125.0 | 24 |
| 51 | p, p’-DDE | 246.0>176.0 | 30 | 317.9>248.0 | 24 |
| 52 | Dieldrin | 276.9>241.0 | 8 | 262.9>193.0 | 34 |
| 53 | o, p’-DDD | 235.0>165.0 | 24 | 237.0>165.0 | 28 |
| 54 | Chlorfenapyr | 247.1>227.0 | 16 | 139.0>102.0 | 12 |
| 55 | Nitrofen | 202.0>139.0 | 24 | 282.9>253.0 | 12 |
| 56 | Endrin | 262.9>191.0 | 30 | 262.9>193.0 | 28 |
| 57 | β-Endosulfan | 194.9>160.0 | 8 | 194.9>125.0 | 24 |
| 58 | p, p’-DDD | 235.0>165.0 | 24 | 237.0>165.0 | 28 |
| 59 | o, p’-DDT | 235.0>165.0 | 24 | 237.0>165.0 | 28 |
| 60 | Endosulfan sulfate | 271.8>236.9 | 18 | 386.8>252.9 | 16 |
| 61 | p, p’-DDT | 235.0>165.0 | 24 | 237.0>165.0 | 28 |
| 62 | Bifenthrin | 181.1>166.1 | 12 | 181.1>179.1 | 12 |
| 63 | Bromopropylate | 340.9>182.9 | 18 | 340.9>184.9 | 20 |
| 64 | Methoxychlor | 227.1>169.1 | 24 | 227.1>212.1 | 14 |
| 65 | Fenpropathrin | 181.1>152.1 | 22 | 265.1>210.1 | 12 |
| 66 | Phenothrin-1 | 123.1>81.0 | 8 | 183.1>153.1 | 14 |
| 67 | Phenothrin-2 | 123.1>81.0 | 8 | 183.1>153.1 | 14 |
| 68 | Cyhalothrin-1 | 208.0>181.0 | 8 | 197.0>141.0 | 12 |
| 69 | Acrinathrin | 181.1>152.1 | 26 | 289.1>93.0 | 14 |
| 70 | Cyhalothrin-2 | 208.0>181.0 | 8 | 197.0>141.0 | 12 |
| 71 | Mirex | 271.8>236.8 | 18 | 273.8>238.8 | 18 |
| 72 | Acrinathrin-2 | 181.1>152.1 | 26 | 289.1>93.0 | 14 |
| 73 | Permethrin-1 | 183.1>153.1 | 14 | 183.1>168.1 | 14 |
| 74 | Permethrin-2 | 183.1>153.1 | 14 | 183.1>168.1 | 14 |
| 75 | Cyfluthrin-1 | 163.1>127.1 | 6 | 163.1>91.0 | 14 |
| 76 | Cyfluthrin-2 | 163.1>127.1 | 6 | 163.1>91.0 | 14 |
| 77 | Cyfluthrin-3 | 163.1>127.1 | 6 | 163.1>91.0 | 14 |
| 78 | Cyfluthrin-4 | 163.1>127.1 | 6 | 163.1>91.0 | 14 |
| 79 | Cypermethrin-1 | 163.1>127.1 | 6 | 163.1>91.0 | 14 |
| 80 | Cypermethrin-2 | 163.1>127.1 | 6 | 163.1>91.0 | 14 |
| 81 | Cypermethrin-3 | 163.1>127.1 | 6 | 163.1>91.0 | 14 |
| 82 | Flucythrinate-1 | 199.1>157.1 | 10 | 157.1>107.1 | 12 |
| 83 | Quizalofop-ethy-l | 372.1>299.1 | 14 | 299.1>255.1 | 18 |
| 84 | Cypermethrin-4 | 163.1>127.1 | 6 | 163.1>91.0 | 14 |
| 85 | Flucythrinate-2 | 199.1>157.1 | 10 | 157.1>107.1 | 12 |
| 86 | Fenvalerate-1 | 225.1>119.1 | 20 | 225.1>147.1 | 10 |
| 87 | Fenvalerate-2 | 225.1>119.1 | 20 | 225.1>147.1 | 10 |
| 88 | Deltamethrin-1 | 180.9>151.9 | 22 | 252.9>93.0 | 20 |
| 89 | Deltamethrin-2 | 180.9>151.9 | 22 | 252.9>93.0 | 20 |

Notes: (1) Compound 29 is the internal standard compound.

(2) Some pesticides have multiple retention time because of isomers.

**Table S2**  Quantitative analysis methodological results of the three target pesticides that were screened. (n=6)

| pesticides | Linear regression equation | R^2^ | LOD(μg/L) | LOQ(μg/L) | RSD for inter-day (%) | RSD for intra-day (%) |
| --- | --- | --- | --- | --- | --- | --- |
| Chlorpyrifos | Y = 402X + 3.55×10^3^ | 0.9991 | 0.0225 | 0.0964 | 1.97 | 2.98 |
| Fipronil | Y = 291X - 1.38×10^3^ | 0.9996 | 0.0155 | 0.0565 | 1.44 | 3.17 |
| Procymidone | Y = 594X + 2.91×10^3^ | 0.9993 | 0.0088 | 0.0354 | 1.63 | 3.82 |
